# COVID-19 control measures implemented by dentists in a northwestern municipality of São Paulo, Brazil

**DOI:** 10.3205/dgkh000557

**Published:** 2025-06-16

**Authors:** Renan Lemos Silva, Monica Moreno Carvalho, Luciana Estevam Simonato

**Affiliations:** 1 Department of Oral Biology, Bauru School of Dentistry, University of São Paulo, Bauru, Brazil; 2 Department of Stomatology, Araçutuba School of Dentistry, State University of São Paulo, Araçatuba, Brazil; 3 Dentistry School, Brazil University, Fernandópolis, Brazil

**Keywords:** COVID-19, dentistry, personal protective equipment, hand hygiene

## Abstract

**Introduction:**

The COVID-19 pandemic posed unprecedented challenges to public health, necessitating strict biosafety measures to mitigate virus transmission. In the dental field, professionals faced heightened risks due to direct contact with aerosols and bodily fluids during clinical procedures. This study aimed to analyze the control measures adopted in 2020 by dental surgeons in the municipality of Fernandópolis, located in northwestern São Paulo State, Brazil, focusing on the adequacy of biosafety practices in Basic Health Units (UBS).

**Results:**

The study found high adherence to the use of personal protective equipment, such as N95 masks, face shields, and disposable medical gowns, alongside rigorous hand-hygiene practices. However, initial difficulties in accessing supplies and raising patient awareness about preventive measures were reported.

**Conclusion:**

Although dental surgeons implemented effective measures to minimize contamination risks, continuous training and awareness strategies remain essential to enhance protection in dental settings.

## Zusammenfassung

**Einleitung:**

Die COVID-19-Pandemie stellte die öffentliche Gesundheit vor beispiellose Herausforderungen und erforderte strenge Biosicherheitsmaßnahmen zur Eindämmung der Virusübertragung. Im zahnmedizinischen Bereich waren Fachkräfte aufgrund des direkten Kontakts mit Aerosolen und Körperflüssigkeiten während klinischer Eingriffe besonders gefährdet. Die Studie zielte darauf ab, die von Zahnärzten im Jahr 2020 in der Stadt Fernandópolis im Nordwesten des Bundesstaates São Paulo, Brasilien, ergriffenen Schutzmaßnahmen mit dem Schwerpunkt der Angemessenheit der Biosicherheitspraktiken in den Basisgesundheitsstationen (UBS’s) zu analysieren.

**Ergebnisse:**

Es zeigte sich eine hohe Adhärenz zur Nutzung persönlicher Schutzausrüstung wie N95-Masken, Gesichtsschutzschirme und Einwegkittel sowie zur Einhaltung strenger Händehygiene. Es wurden jedoch anfängliche Schwierigkeiten beim Zugang zu Materialien und bei der Sensibilisierung der Patienten für Präventionsmaßnahmen berichtet.

**Schlussfolgerung:**

Obwohl Zahnärzte bedeutende Maßnahmen zur Minimierung von Kontaminationsrisiken umgesetzt haben, sind kontinuierliche Schulungs- und Sensibilisierungsstrategien weiterhin unerlässlich, um den Infektionsschutz im zahnmedizinischen Bereich zu stärken.

## Introduction

Shortly after the declaration of the COVID-19 pandemic on March 11, 2020 by the World Health Organization (WHO) [1], new containment measures were quickly implemented to reduce mass population infections. These measures included social distancing and isolation, which caused behavioral changes worldwide [2], [3].

The emergence of the novel respiratory pathogen SARS-CoV-2 highlighted the importance of using personal protective equipment (PPE) among healthcare professionals to prevent viral infection [4]. This equipment was to be used based on the risk of exposure and the transmission dynamics of the pathogen. For clinical care, healthcare professionals providing direct patient care should wear gloves, medical masks, and eye protection. The management of procedures that generate aerosols – such as tracheal intubation, non-invasive ventilation, tracheostomy, cardiopulmonary resuscitation, manual ventilation before intubation, and bronchoscopy – requires the use of respirators, gloves, and gowns. Additionally, aprons should be used if gowns are not fluid-resistant [5]. Individuals with respiratory problems or symptoms, as well as those caring for COVID-19 patients at home, should use medical masks. For asymptomatic individuals, adherence to state and municipal guidelines was essential to align with appropriate periods of isolation and social relaxation measures [2].

Given the current circumstances, new services related to safety measures have been adopted to ensure greater protection for both healthcare service users and providers. One essential system is the establishment of telemedicine laboratories, enabling blood tests, result validation, diagnoses, and various tasks through a networked system connecting multiple hospitals into a single virtual unit [6].

Although national and international authorities have not officially classified the risk level of COVID-19, a global consensus has been reached, designating it as Biosafety Level 3 (BSL-3) due to its high pathogenicity, virulence, and prolonged viability on objects or surfaces [7]. According to the Evandro Chagas National Institute of Infectiology in 2020, BSL-3 measures for workers exposed to the virus included standard practices such as hand antisepsis before and after handling viable materials, before and after glove use, and after handling infectious materials even when wearing gloves. PPE measures included wearing specific clothing such as coveralls, caps, masks (N95 masks recommended for aerosol-generating procedures), and gloves, which should be autoclaved before washing or discarded. Additional measures included signage indicating biological risk, patient isolation, and organization of transit areas within the facility.

In healthcare settings, after identifying a suspected case of influenza-like syndrome, patients should be provided with surgical masks in the waiting area, even before triage, and directed to a specific room for respiratory isolation. These rooms should remain closed, without air conditioning, and with windows always open [8].

For dental professionals, as recommended by the Ministry of Health, protection follows the same principles mentioned above, with the addition of PPE such as surgical masks, caps, gloves, protective eyewear, face shields, and disposable long-sleeved impermeable gowns. When performing aerosol-generating procedures, dentists must wear N95/PFF2 face respirators [9], strictly adhering to correct donning and doffing techniques [8]. Thus, the present study aims to identify the COVID-19 pandemic control measures adopted by dental surgeons in a municipality in northwestern São Paulo State in 2020.

## Materials and methods

This study was characterized as a non-experimental, cross-sectional, quantitative, and descriptive study.

### Ethical aspects

The project was approved by the Research Ethics Committee (CEP) of Universidade Brasil under the number CAAE: 39614620.6.0000.5494.

Each participant signed the Informed Consent Form (TCLE), in accordance with Ordinance No. 466/2012.

### Sample

The research was conducted with dental surgeons from the public health network in the city of Fernandópolis, SP.

### Data collection

Data were collected using an electronic questionnaire through a Google platform (Google Forms^®^).

## Results

The questionnaire, administered throughout July 2021 to 32 dental surgeons, yielded 27 (84.4%) responses. Among the respondents, 22 (84.6%) were female and 5 (15.4%) were male. The age distribution was as follows: the <40-year-olds produced 12 responses (46.2%), and among those 30 to 34 years old, there were 3 responses (11.5%). Regarding years from graduation, 12 (46.2%) had graduated 21 years ago or more, followed by 7 (26.9%) who had graduated 6 to 10 years ago, 5 (19.2%) graduated 11 to 15 years ago, and 2 (7.7%) graduated 16 to 20 years ago.

All participants held a degree in dentistry, and some specialists held or did not hold the title of master or doctor. In terms of the highest educational qualification, 23 (73.1%) were specialists, 3 (11.5%) had only an undergraduate degree, 3 (11.5%) had a master’s degree, and 1 (3.8%) held a doctorate. Among the specialists, 10 (38.5%) had a specialization in public health, 5 (19.2%) in orthodontics, 4 (15.4%) in endodontics and public health dentistry, 3 (11.5%) in implantology, pediatric dentistry, and general dentistry, and 1 (3.8%) had a specialization in oral and maxillofacial surgery, restorative dentistry, stomatology, functional jaw orthopedics, dental prosthetics, and dental radiology.

Regarding living arrangements and household dynamics, 18 (69.2%) of the dental surgeons lived with family members (spouse/partner, children), 5 (19.2%) lived with other relatives (parents, grandparents, uncles, etc.), 2 (7.7%) lived alone, and 1 (3.8%) lived with others. Additionally, 18 (69.2%) had children.

Regarding chronic diseases and habits, 3 (11.5%) reported having a chronic condition requiring continuous medication. In terms of habits, 2 (7.7%) were smokers, 2 (7.7%) were former smokers, and 12 (46.2%) reported alcohol consumption. Furthermore, 13 (50%) of the professionals engaged in sports once or twice a week, 7 (26.9%) practiced more than three times a week, and 6 (23.1%) did not practice any sports.

**Figure 1:**
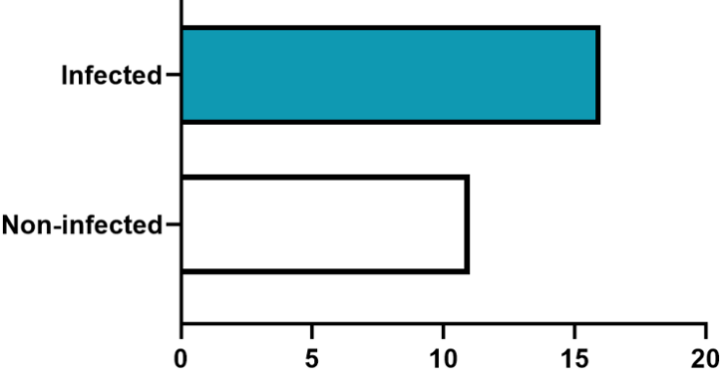
Distribution of professionals based on COVID-19 infection status: 16 were infected, while 11 remained uninfected.

During the COVID-19 pandemic, 3 (11.5%) had traveled in the last 3 months, and 13 (50%) had contact with individuals who had traveled in the past 3 months. Additionally, 16 professionals (59.26%) were infected with COVID-19, while 11 (40.74%) remained uninfected (Figure 1 (Fig. 1)).

The questionnaire asked about the working system adopted, number of patients seen per shift, and age groups of patients treated. Twenty-six 27 (100%) of the professionals worked with a four-handed approach during procedures. The number of patients seen during a 4-hour work shift varied, with 17 (65.4%) seeing 1 to 4 patients, 7 (26.9%) seeing 5 to 8 patients, and 2 (7.7%) seeing 9 or more patients. 19 (73.1%) of the professionals treated both adults and children, 4 (15.4%) saw adults only, and 3 (11.5%) treated children only.

The respondents performed a self-assessment of their knowledge of COVID-19, with 16 (61.5%) reporting sufficient knowledge and 10 (38.5%) reporting moderate knowledge. Information sources regarding the novel coronavirus were obtained from official websites of entities such as the Ministry of Health, WHO, and professional organizations, and/or their social media accounts; 26 (100%) reported using these sources. Additionally, 15 (57.7%) received information from personal websites/accounts of healthcare professionals, 17 (65.4%) from television, 11 (42.3%) from medical books, journals, or articles (printed or online), 8 (30.8%) from events such as seminars, meetings, and institutional congresses, and 14 (53.8%) received updates through communication groups (WhatsApp, Telegram, etc.). Only 1 (3.8%) did not participate in any training provided by their workplace.

**Figure 2:**
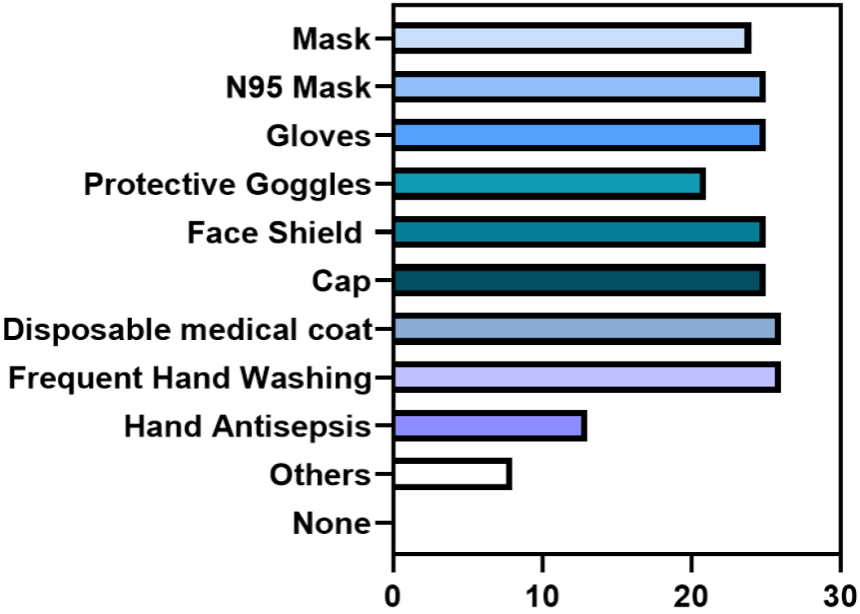
Distribution of data regarding the measures adopted against COVID-19 during work

The dental surgeons implemented various measures in their practice against COVID-19, including the use of masks (24 [92.3%] responders), N95 masks (25 responders [96.2%]), gloves (25 [96.2%]), protective goggles (21 [80.8%]), face shields (25 [96.2%]), caps (25 [96.2%]), disposable medical gowns (26 [100%]), frequent handwashing (26 [100%]), and hand hygiene (26 [100%]). Other measures were adopted by 8 (30.8%) of the professionals (Figure 2 (Fig. 2)).

Several issues were reported by the professionals during the COVID-19 pandemic, including 7 (26.9%) who experienced difficulty accessing personal protective equipment (PPE), 4 (15.4%) who had trouble raising awareness among their professional colleagues, 5 (19.2%) who had difficulty raising awareness among healthcare assistants, 16 (61.5%) who reported low patient awareness, and 7 (26.9%) who reported no issues. As dental surgeons are in an occupational risk group for COVID-19, the concern about transmitting the disease to family members was shared by 24 (92.3%) of the professionals.

**Figure 3:**
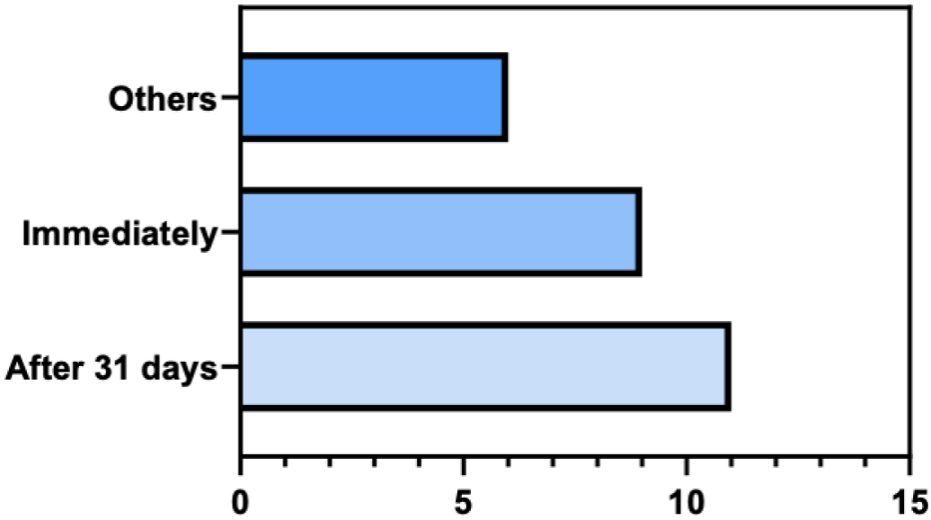
Distribution of data regarding the time lapse before receiving the appropriate PPE for patient care

At the start of the pandemic, professionals faced a delay in receiving appropriate PPE for patient care. 11 (42.3%) reported receiving PPE after 31 days, while 9 (34.6%) said they received them immediately because they already had them (Figure 3 (Fig. 3)).

**Figure 4:**
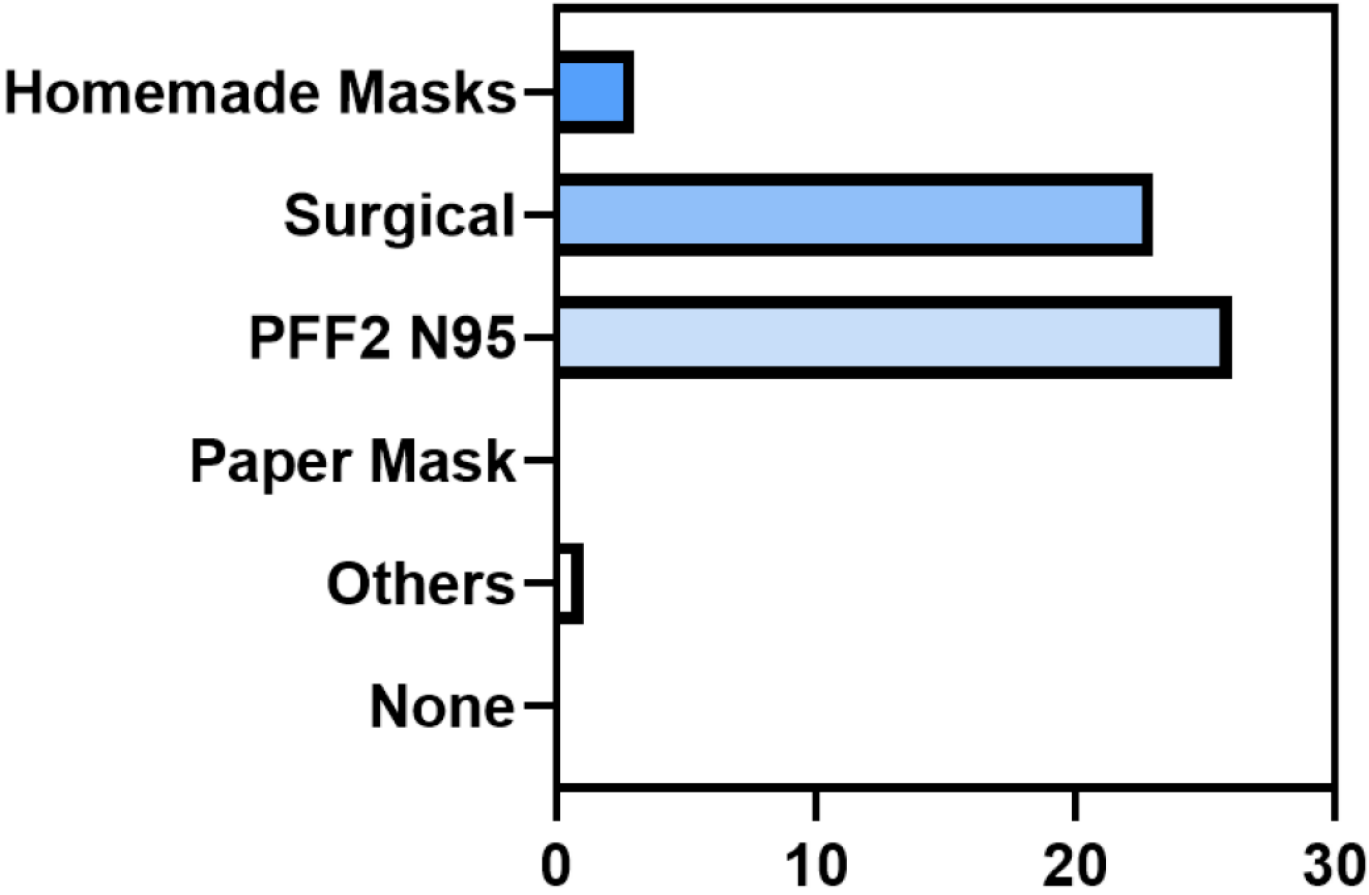
Distribution of data regarding the type of mask received

The PPE received included gloves (26, 100%), masks (26, 100%), caps (26, 100%), protective goggles (24, 92.3%), face shields (26, 100%), and disposable medical gowns (26, 100%). The types of masks received were fabric masks (3, 11.5%), surgical masks (23, 88.5%), and N95 masks (26, 100%) (Figure 4 (Fig. 4)).

Regarding the replacement frequency of N95 masks, 17 (65.4%) reported changing them biweekly, 2 (7.7%) reported changing them monthly, and 7 (26.9%) reported changing them weekly. In terms of disposable medical gowns, 18 (69.2%) changed them daily, 7 (26.9%) changed them weekly, and 1 (3.8%) changed them biweekly.

The provision of PPE in the Basic Health Units (UBS) did not present difficulties for 25 (96.2%) of the dentists. However, 4 (15.4%) reported occasional shortages of PPE at the UBS. According to the respondents, there was no shortage of either solid or liquid soap or hand sanitizer (alcohol gel or 70% alcohol) for hand hygiene at any time.

During the pandemic, various measures were adopted by the professionals, such as “leaving shoes outside the home”, which was done by 20 (76.9%); “taking a shower before doing any other activity”, reported by 21 (80.8%); “leaving contaminated clothes in the laundry”, done by 21 (80.8%); “washing hands with soap and water or using alcohol gel”, done by 26 (96.2%); and “disinfecting personal items”, done by 16 (61.5%).

All respondents underwent COVID-19 diagnostic testing, with 19 (73.1%) undergoing immunological tests and 21 (80.8%) undergoing PCR-RT tests. The results were 20 (76.9%) negative and 6 (23.1%) positive.

Regarding mental health, 18 (69.2%) reported feeling psychologically affected, while 8 (30.8%) did not feel affected. In terms of safety in dealing with the COVID-19 pandemic, 13 (50%) felt secure, while 13 (50%) did not feel secure.

## Discussion

The COVID-19 pandemic presented numerous challenges and a high risk of contamination for healthcare professionals, particularly for dental surgeons, who face a significantly higher occupational risk. This heightened risk stems from their close, face-to-face work with patients, involving direct contact with the oral cavity, saliva, blood, and aerosols produced during most procedures [7], [10]. Public healthcare professionals in Fernandópolis, São Paulo, demonstrated openness to training and adapting to this new reality.

According to the retrospective study by Thomé et al. [9], multiple authors emphasize the importance of hand hygiene and the use of PPE, including gloves, surgical masks, N95 masks, caps, disposable medical coat, and face shields. In our study, we observed the frequency of use of these protective measures and their availability as provided by the local health department.

The World Health Organization [1] mandates that healthcare professionals must use respiratory protection masks (particulate respirators) with a minimum filtration efficiency of 95% for particles as small as 0.3 µm, such as N95, N99, N100, PFF2, or PFF3 masks. During the initial phase of the pandemic, N95 masks were distributed to healthcare workers and were reused for a period of 15 days, with proper removal and storage, as reported by most respondents. This aligns with guidance from the Brazilian National Health Surveillance Agency (ANVISA, in Portuguese *“Agência nacional de vigilância Sanitária”*), which states that N95 or equivalent masks may be reused by the same professional if mandatory steps are followed to remove the mask without contaminating its interior [11].

Additionally, face shields were distributed to protect against aerosols generated during procedures, with 25 respondents (96.2%) reporting regular use of this protective equipment. Face shields can reduce contamination of the N95 mask or equivalent. When available, a face shield can be used, and if the mask remains intact, clean, and dry, it may be reused multiple times during the same treatment period by the professional.

## Conclusion

Various COVID-19 pandemic control measures were adopted by dental surgeons in the municipality of Fernandópolis, São Paulo, to reduce the risk of contamination among healthcare professionals while maintaining dental services.

## Notes

### Ethical approval

The project was approved by the Research Ethics Committee of Universidade Brasil under the number CAAE: 39614620.6.0000.5494.

### Funding

None. 

### Acknowledgments

We acknowledge the support of the Municipal Government of Fernandópolis-SP, Brazil, and the Unified Health System (SUS). 

### Authors’ ORCIDs

- Renan Lemos da Silva: 0000-0001-5837-410X
- Monica Moreno Carvalho: 0000-0002-2579-2951
- Luciana Estevam Simonato: 0000-0002-6413-5479

### Competing interests

The authors declare that they have no competing interests.
